# Genetic Variants in Epidermal Differentiation Complex Genes as Predictive Biomarkers for Atopic Eczema, Allergic Sensitization, and Eczema-Associated Asthma in a 6-Year Follow-Up Case–Control Study in Children

**DOI:** 10.3390/jcm11164865

**Published:** 2022-08-19

**Authors:** Anna Dębińska, Hanna Danielewicz, Barbara Sozańska

**Affiliations:** Department and Clinic of Paediatrics, Allergology and Cardiology Wrocław Medical University, ul. Chałubińskiego 2a, 50-368 Wroclaw, Poland

**Keywords:** atopic eczema, atopic march, asthma, hornerin, filaggrin-2, filaggrin, mutations, biomarkers, genetic association, skin barrier

## Abstract

Atopic eczema is the most common chronic inflammatory skin disease of early childhood and is often the first manifestation of atopic march. Therefore, one challenge is to identify the risk factors associated with atopic eczema that may also be predictors of atopic disease progression. The aim of this study was to investigate the association of SNPs in hornerin (HRNR) and filaggrin-2 (FLG2) genes with childhood atopic eczema, as well as other atopic phenotypes. Genotyping for HRNR and FLG2 was performed in 188 children younger than 2 years of age, previously screened for the FLG null mutations, and followed at yearly intervals until the age of 6. We demonstrated that risk variants of HRNR rs877776[C] and FLG2 rs12568784[T] were associated with atopic eczema, allergic sensitization, and susceptibility to the complex phenotype—asthma plus eczema. These effects seem to be supplementary to the well-known associations for FLG mutations and may be modulated by gene–gene interactions. Additionally, in children with eczema, these genetic variants may also be considered, along with FLG mutations, as predictive biomarkers for eczema-associated asthma. In conclusion, our results indicate that genetic variants in the epidermal differentiation complex gene could contribute to the pathogenesis of atopic eczema and progression to subsequent allergic disease.

## 1. Introduction

Atopic eczema is the most common chronic inflammatory skin disease worldwide that predominantly affects children (from 15% to 30% in this age group) [1,2,3]. Moreover, atopic eczema is often the first manifestation of the so-called atopic march and is considered the entry point for later development of other atopic manifestations, including asthma and allergic rhinitis [4,5,6,7]. One of the most intriguing targets in current research efforts is to identify the risk factors associated with atopic eczema that may also be predictors of atopic disease progression. Therefore, a better understanding of atopic eczema pathogenesis seems to be extremely important in the context of the prevention of allergic diseases. Atopic eczema is a heterogeneous disease with a complex and multifactorial background, including interactions between genetic susceptibility and environmental factors [8,9]. According to the “outside-to-inside” theory, the epidermal barrier dysfunction is recognized as the primary defect and crucial determinant of atopic eczema pathogenesis [10,11]. Moreover, recent studies have demonstrated that skin barrier impairment is not only crucial for the development of atopic eczema, but also other allergic disorders [12,13,14]. Though our understanding of the underlying mechanisms of these abnormalities remains limited, the most widely established cause for skin barrier dysfunction involves genetically determined downregulation of epidermal differentiation complex (EDC) proteins, especially filaggrin (FLG) [15,16,17]. Indeed, FLG loss-of-function mutations are known to be the strongest and most widely replicated genetic risk factors associated with atopic eczema [18,19,20,21]. In addition, subsequent reports have suggested an association of the FLG null mutations with other allergic diseases, especially asthma in the context of eczema [22,23,24,25]. Nevertheless, the FLG gene is only one of 70 genes located in the EDC on chromosome 1q21; genetic and experimental studies have indicated that FLG mutations may not be solely responsible for the strong genetic association between this region and atopic eczema [1,18,26,27]. FLG mutations are found in, at most, one-half of atopic eczema cases, even in the cohorts with the most severe cases [20,28,29,30]. Additionally, impaired skin barrier functions were demonstrated in atopic eczema patients irrespective of their FLG genotypes, suggesting that other genes within the EDC region could also contribute to epidermal barrier dysfunction and may be involved in the pathogenesis of atopic eczema and progression to subsequent allergic disease [31,32,33]. Importantly, recent GWASs have identified the additional association signals connected with atopic eczema within EDC apart from the mutations in the FLG gene [34,35,36,37,38].

The EDC is a cluster of several gene families encoding structural and regulatory proteins primarily expressed in the epidermis that contribute to the formation of the cornified envelope (CE), terminal keratinocyte differentiation, and proper skin barrier function [39,40]. One of these EDC gene families encodes the “fused gene” proteins (SFTPs ‘S-100 fused type protein’), such as filaggrin, filaggrin-2, hornerin, trichohyalin, repetin, and cornulin, which are the major cytoplasmic matrix proteins cross-linked to the CE and, thus, are substantial for proper keratinocyte cornification and stratum corneum properties [41]. Two interesting proteins, hornerin (HRNR) and filaggrin-2 (FLG2), show numerous similarities to FLG, such as colocalization in the epidermis, high sequence homology in the molecular structure, analogous amino acid composition, as well as the same physicochemical properties, indicating that these proteins could be functionally related to FLG [42]. Indeed, experimental studies suggested that FLG-2 and HRNR may be similar or complementary to FLG’s role in the process of cornification and formation of the skin barrier [43,44,45]. Furthermore, the expression levels of HRNR and FLG-2 were shown to be significantly reduced in the skin of patients with atopic eczema, as was also demonstrated for FLG [32,46,47]. Based on these findings, it seems reasonable to assume that genetic variants in HRNR and FLG-2 genes might contribute to the pathogenesis of atopic eczema by possible influence on the gene expression or protein function.

In the current study, we assessed the importance of SNPs in HRNR and FLG2 genes in the susceptibility to childhood atopic eczema and whether this possible association is independent of the FLG risk alleles described previously in this population [48]. Moreover, we evaluated the association between these risk variants with the severity of atopic eczema and other atopic phenotypes, such as allergic sensitization and eczema-associated asthma.

## 2. Materials and Methods

### 2.1. Study Population

A total of 188 unrelated children (107 males) younger than 2 years old at the time of recruitment were enrolled, namely 103 patients with atopic eczema (mean age 13.2 ± 6.7 months) and 85 normal control subjects (mean age 15.3 ± 5.6 months). The whole study population was followed at yearly intervals until age 6. All study participants were of Caucasian ethnicity. The study subjects were recruited from patients who visited the Outpatient Clinic for Children at Wroclaw Medical University Hospital and from the general population, as described below. The healthy control subjects were recruited from the general population through community-based approaches. We distributed flyers at local nurseries, child and family doctor surgeries, and health fairs. Interested parents were instructed to contact the research coordinator via phone. All participants, cases, and controls, were selected using a detailed questionnaire that included questions regarding the overall health status, symptoms of atopic eczema and other allergic diseases, sociodemographic information, and family histories of allergic diseases. The subjects with atopic eczema were examined and diagnosed according to the criteria established by Hanifin and Rajka [49]. The mean age at disease onset was 4.6 ± 3.5 months. Atopic eczema disease severity was assessed by using the SCORing Atopic Dermatitis index (SCORAD) and the patients were divided into mild (<25 points), moderate (25–50 points), or severe (>50 points) disease groups. Subjects with atopic eczema were divided into allergic and nonallergic on the basis of positive specific IgE levels against at least one of the allergens tested at the time of recruitment. “Asthma was defined as physician-diagnosed asthma ever by the age 6 years. Asthma at 6 years of age was defined by the presence of a previous asthma diagnosis made by doctor during the follow-up visits and 1 or more wheezing episodes during the 12 months before the analysis. Eczema-associated asthma at the 6 years of age was defined by the presence of previous physician’s diagnosis of eczema according to the criteria established by Hanifin and Rajka and visible eczema at the time of follow-up together with the presence of a previous asthma diagnosis made by doctor during the follow-up visits and 1 or more wheezing episodes during the 12 months before the analysis”. The control group (matched with the case for age and gender) included healthy children and met the following criteria: absence of symptoms of atopic eczema and asthma and negative family history of allergic diseases.

In all recruited subjects, serum measurements for total and specific IgE levels were performed, including IgE specific for the 10 most popular inhalants (Dermatophagoides pteronyssinus, Dermatophagoides farina, cat, dog, horse epithelia, Birch pollen, Timothy Grass pollen, Mugwort pollen, Aspergillus fumigatus, Cladosporium herbarum) and 10 food allergens (peanut, milk, egg white, egg yolk, potato, carrot, cod, apple, soya, wheat flour). The concentration of total serum IgE was measured by using the commercially available kit IMMULITE 2000 Total IgE (Diagnostic Products Corporation (DPC), Los Angeles, CA, USA). The levels of specific IgE were determined using a standard enzyme immunoassay (Polycheck, Biocheck GmbH, Munster, Germany). Allergic sensitization was defined as the presence of specific IgE (to at least one of the tested allergens) of ≥0.7 kU/L (class II). Polysensitization was defined as the presence of a specific IgE level of ≥0.7 kU/L (class II) or greater to more than one tested allergen.

### 2.2. Genotyping

The samples of the 188 subjects were genotyped for HRNR rs877776 and FLG2 rs12568784 SNPs. The whole blood samples for genetic testing were collected at the time of recruitment, before the age of 2, and the genotyping was conducted immediately thereafter. The study populations were also genotyped for the 4 common FLG mutations: R501X, 2282del4, R2447X, and S3247X, as described previously [48]. Genomic DNA was obtained from EDTA whole blood samples using the QIAamp DNA Blood Mini Kit (QIAGEN GmbH, Germany). All mutations were determined by using the LightSNiP assay (TibMolbiol, Berlin, Germany). PCR was performed at a final volume of 10 µL containing 1 µL of DNA at a concentration of 15–60 ng/µL, 0.5 µL of reagent mix containing specific primers and SimpleProbes^®^ probesat optimized concentration, 0.8 µL of MgCl2, and 1 µL of LightCycler^®^FastStart DNA MasterHybProbe (Roche Applied Science, Mannheim, Germany). Reactions were performed on a LightCycler 1.5 platform (Roche Applied Science, Mannheim, Germany). For quality control of genotyping procedures, positive and negative controls of each genotype were included in each reaction.

### 2.3. Statistical Analysis

The Hardy–Weinberg equilibrium was tested using the χ^2^ goodness-of-fit test to compare the observed genotype frequencies with the expected frequencies among the controls. Differences in genotype frequencies or demographic characteristics between case and control groups were evaluated using the χ^2^ test or Fisher exact test, as appropriate. The associations of genotypes or alleles with patient groups versus control subjects were determined by computing the odds ratio (OR), 95% confidence interval (95%CI), and *p* values using a logistic regression analysis for crude ORs and adjusted ORs when adjusting for age, gender, and family history of atopy. Statistical significance was set at a *p* value < 0.05. Two different genetic models were tested separately when comparing genotypes and disease phenotypes in this study. To test the dominant model, wild type homozygotes were compared with heterozygotes and homozygotes for minor alleles. In the multiplicative model, genotypes were coded as 3-level variables for the minor allele numbers. χ^2^ or Fisher exact tests were used to determine the combined effects of genotype pairs. Gene–gene interactions were investigated by using logistic regression models for atopic eczema with interaction terms (SPSS). To establish whether an interaction between the two risk factors A and B existed, the relative excess risk due to interaction (RERI), the proportion attributable to interaction (AP), the synergy index (S), and the ratio of ORs were calculated, as recommended by Knoll et al. [50,51]. Interactions were defined as departures from the additive or multiplicative models. On an additive scale, a RERI and an AP > 0 or <0 indicate superadditive or subadditive effects, respectively, S > 1 or <1 mean superadditive or subadditive effects, respectively. On the multiplicative scale, the ratios of ORs >1 or < 1 indicate positive or negative interactions, respectively. The predictive values of risk factors were assessed through analyses of the sensitivity, specificity, and positive and negative predictive values. The statistical analyses were carried out using the program package STATISTICA Version 9.0 (StatSoft, Inc., Tulsa, OK, USA) and the SPSS Statistics software package Version 11.1 (SPSS Inc., Chicago, IL, USA).

The study was approved by the ethics committee of Wroclaw Medical University, Wroclaw, Poland (protocol codes: 392/2011 and 631/21) and informed written consent (including consent for genetic studies) was obtained for all of the subjects before testing.

## 3. Results

A summary of characteristics of the patients with atopic eczema and the controls, including the genotyping results for HRNR rs877776 and FLG2 rs12568784 SNPs, as well as the distribution of the FLG genotype, are presented in Table 1. There were no significant differences between the cases and the controls for age and gender.

### 3.1. The Association of HRNR rs877776 and FLG2 rs12568784 with Eczema

HRNR rs877776 SNP showed a significant association with atopic eczema (*p* = 0.013). The OR associated with rs877776 SNP was estimated at 1.99 (95%CI 1.12–3.56) by using the allele model. In the dominant model adjusted for potential confounders, [C] allele was associated with a significantly increased risk for atopic eczema (OR = 2.48; 95%CI 1.25–4.99; *p* = 0.005) compared with the wild type homozygote (Table 2A). There were no significant associations between the HRNR rs877776 SNP and atopic eczema severity. The mild and moderate atopic eczema groups did not differ in terms of frequency of HRNR rs877776 SNP (*p* = 0.879; χ^2^ = 0.259) (Table 2A).

Analysis of the FLG2 rs12568784 polymorphism revealed a significant association with susceptibility to atopic eczema (for allele OR = 1.91; 95%CI 1.01–3.65; *p* = 0.003). Patients carrying at least one allele T for FLG2 SNP were two times more likely to have atopic eczema when compared to patients who did not carry any of these variants (Table 3A). Furthermore, the multivariate analysis of the genotypes adjusted for age, sex, and family history of atopy confirmed these associations. We also observed a highly significant association between the FLG2 SNP with disease severity; a patient carrying at least one FLG2 rs12568784 allele [T] was approximately four times more likely of having moderate atopic dermatitis (Table 3A).

Next, we investigated whether the strongly-significant null mutations in the FLG gene, which is also located within EDC, influenced our results in eczema cases. The FLG mutations were identified in this population previously and the combined genotypes of four mutations were significantly associated with AE. Additional analyses performed after stratification for the presence or absence of the combined FLG mutations indicated the significant association between HRNR rs877776 risk allele [C] and atopic eczema in the group without FLG mutations. The FLG2 rs12568784 variant analysis after stratification showed a statistically significant effect only on the presence of strongly significant FLG mutations (Table 4). Therefore, we included the combined FLG genotype as a second predictor in a logistic regression model to determine whether the FLG mutations confounded the observed associations. After adjusting for the presence of FLG mutations, the HRNR rs877776 still showed a statistically significant effect (*p* = 0.005) with an OR of 2.51 (95%CI 1.30 ÷ 4.83), while the effects of the FLG2 polymorphism did not remain significant (OR = 1.65; 95%CI 0.76–3.57; *p* = 00201).

### 3.2. The Effects of HRNR rs877776 and FLG2 rs12568784 on Eczema-Associated Asthma

The case–control study design enabled us to evaluate the association between HRNR and FLG2 SNPs and asthma in the overall population because all cases of physician diagnoses of asthma by the age of 6 were observed in patients with atopic eczema; therefore, all our patients with asthma represented the complex phenotype—asthma plus eczema. However, we revealed that the risk alleles HRNR rs877776[C] and FLG2 rs12568784[T], predisposed to eczema-associated asthma, significantly increased the risk of this combined phenotype by nearly four times and more than five-fold, respectively (Table 2A and Table 3A). Next, to test whether this increased risk was mainly due to the association for eczema or reflected the real effects of these risk alleles on eczema-associated asthma, we restricted the analysis to children with eczema and evaluated the association of HRNR and FLG2 SNPs with the eczema-associated asthma phenotype. Interestingly, we confirmed significant associations between both HRNR and FLG2 risk alleles and asthma in this subgroup (Table 2B and Table 3B).

### 3.3. The Effects of HRNR rs877776 and FLG2 rs12568784 on Allergic Sensitization

We demonstrated significant associations of HRNR rs877776 and FLG2 rs12568784 risk alleles with IgE-mediated sensitization in the overall population. These associations with sensitization to common allergens were slightly more pronounced for multiply-sensitized children (Table 2A and Table 3A). Next, we restricted the analysis to children with eczema and demonstrated a significant association between HRNR and FLG2 SNPs and allergic sensitization in this subgroup (Table 2B and Table 3B). Interestingly, the effects of HRNR rs877776 and FLG2 rs12568784 risk alleles on allergic sensitization were also independent of concomitant allergic diseases, as the associations were significant in the subgroup of controls without eczema and asthma (OR = 9.04; 95%CI 2.28 ÷ −35.76; *p* = 0.002).

### 3.4. Analysis of Interaction between HRNR rs877776 and FLG2 rs12568784 and FLG Mutations

Moreover, we investigated whether an interaction between HRNR and FLG2 SNPs and the four most common FLG mutations affected the atopic eczema risk. When the HRNR rs877776 variant and FLG mutations were considered together, the risk of atopic eczema was increased the most in subjects who combined at least one rs877776[C] allele and at least one loss-of-function FLG mutation, compared with the reference group, where the children were homozygous for the rs877776[G] allele and non-carriers of FLG mutations. These results indicated multiplicative interactions, as the measure of interaction on a multiplicative scale, the ratio of RRs, was 0.85, meaning that the combined effect was higher than the product of the individual effect. Importantly, the HRNR variant or FLG mutation alone caused a significantly increased risk for eczema (Table 5). Further interaction analyses of these two risk variants with eczema-associated asthma showed even stronger effect sizes for the different risk groups. In addition, we noted the synergistic effects of rs877776[C] and FLG mutations on eczema-associated asthma (RERI = 2.19) (Table 6).

The combined analysis for FLG2 and FLG variants demonstrated that, compared to the reference group carrying neither genetic risk factor, the strong effect was revealed only in subjects who carried both risk factors; the presence of at least one FLG2 rs12568784 [T] allele and at least one loss-of-function FLG mutation significantly increased the atopic eczema risk. The RERI, AP, and synergy index indicated a positive interaction on the additive scale, meaning that the risk conferred by the combination of both risk factors was significantly higher than the sum of the independent effects. We also demonstrated that the risk of eczema in children carrying both risk variants was well-fit by the multiplicative model with the ratio of RRs being 1.45 (Table 5). The evidence for interaction on additive and multiplicative scales was also found in the further analysis regarding the eczema-associated asthma phenotype (RERI = 0.36; ratio of RRs = 0.43) (Table 6).

Finally, the significant association between both risk factors with atopic eczema and eczema-associated asthma prompted us to determine the interactive effects of HRNR and FLG2 SNPs. The presence of both risk factors, at least one HRNR rs877776[C] allele and at least one FLG2 rs12568784 [T] allele, strongly enhanced the risk of atopic eczema. Interestingly, the HRNR variant alone caused a significantly increased risk for atopic eczema, whereas the FLG2 [T] variant provided no additional disease risk in the absence of the HRNR [C] allele (Table 5). In the case of eczema-associated asthma, both HRNR and FLG2 variants alone yielded significantly increased risks; however, the strongest effect was seen in the subjects who combined both at least one HRNR rs877776[C] allele and at least one FLG2 rs12568784 [T] (Table 6). These findings pointed to interaction on the multiplicative scale between HRNR and FLG2 variants in the case of atopic eczema and eczema-associated asthma.

Our results suggest that there is evidence of a gene–gene interaction on an additive and/or multiplicative scale, although when modeling the interaction, the interaction coefficients were not significant for both genotype combinations, which may be because of the low sample size of our study and insufficient statistical power.

### 3.5. HRNR rs877776 and FLG2 rs12568784 in the Prediction of Asthma

Finally, we investigated whether HRNR rs877776 and FLG2 rs12568784 risk variants can be used as predictive biomarkers for the development of eczema-associated asthma in young children. In our population, HRNR rs877776[C] or FLG2 rs12568784[T] alone were modestly strong predictors with positive predictive values of 45.7% and 53.6%, respectively; this indicates that nearly half of the eczema cases who carried the HRNR or FLG2 risk variants will develop eczema-associated asthma phenotype. Next, we added FLG mutations to the prediction model because our previous study in this population revealed that the FLG mutations were strong predictors for eczema-associated asthma, with highly positive predictive values of 66.7% [48], which was also confirmed here. Interestingly, the combination of any tested risk variants, HRNR rs877776[C] or FLG2 rs12568784[T] with FLG mutations, provided the best combination of diagnostic specificity (100%) and predicted eczema plus the asthma phenotype with a positive predictive value of 100% (Table 7).

## 4. Discussion

The aim of our current study was to assess the association between the susceptibility to childhood atopic eczema as well as eczema-associated asthma and genetic variants in the HRNR and FLG2 genes, two worthy investigation members of the S-100-fused type protein family, which also includes FLG. We demonstrated that risk variants of HRNR rs877776 [C] and FLG2 rs12568784[T] were associated with atopic eczema, allergic sensitization, and susceptibility to the complex phenotype—asthma plus eczema. Interestingly, these effects seem to be supplementary to the well-known associations for FLG mutations and may be modulated by gene–gene interactions. Additionally, in children with eczema, these genetic variants, along with FLG mutations, considerably increased the risk of disease progression to eczema-associated asthma.

Over the last decade, evidence has supported the primary and central roles of epidermal barrier dysfunction in atopic eczema pathogenesis [10,11,17,52]. Most of the barrier defects that have been consistently observed in atopic eczema originate in the stratum corneum, particularly in CE, which is essential for the proper functioning of the skin barrier [53,54]. At the molecular level, the downregulated expression of epidermal barrier-related proteins has been extensively highlighted [15,16,17,52,55]. The causes of these abnormalities in the skin barrier are multifactorial and determined by a complex interplay between genetic, immunological, and environmental factors [8,9,53,54]. Among the 34 specific genomic regions identified as associated with atopic eczema susceptibility, the FLG loss-of-function mutations remain the strongest and most replicated risk factors reported to date [18,19,21,22,56]. Our former studies on these populations also provided highly significant replications of the previously reported associations between the four nonsense FLG mutations with atopic eczema [48]. The strengths of the associations attributed to the FLG mutations are extraordinary; however, these genetic variants cannot explain all of the skin barrier abnormalities found in atopic eczema patients [1,9,26]. Therefore, it is tempting to propose that other genes in EDC-encoding CE precursor molecules could also be related to the atopic eczema pathogenesis [57,58]. While the debate is ongoing, GWASs have identified an additional association signal in the EDC on chromosome 1q21 apart from the mutations in the FLG gene [37,59,60,61].

One of the variants we studied was SNP (rs877776), located within the region of the HRNR gene-encoding human hornerin, which has been previously identified in the first GWAS of atopic eczema as a novel independent susceptibility locus for this disease [37,62]. Additionally, the strong association between risk allele C of rs877776 and atopic eczema cases was revealed in further association analyses in all discovery sets [37]. In contrast, the case–control study in the Irish pediatric population with moderate-to-severe eczema did not replicate these results [63]. In our study, we confirmed the association between the C allele of rs877776 and atopic eczema in a population of Polish children, providing replication evidence for this genetic association. However, our analysis showed a little bit higher OR for risk allele [C] carriers compared to that calculated for the discovery cohorts (1.99 vs. 1.06). This may suggest that rs877776 [C] confers a stronger effect in young children because, unlike in our studied population, the population studied by Esperza-Gordillo et al. represents adult cases [37]. Given that the HRNR protein has many structural and functional similarities to FLG, abnormalities in HRNR expression could be involved in the skin barrier dysfunction in atopic eczema [42]. The HRHR protein is expressed in epidermal keratinocytes of healthy skin and is one of the CE components of the human epidermis involved with a late step in CE formation [45,64]. It has been proven experimentally that HRNR is likely responsible for reinforcing the CE and the mechanical properties of the cornified layer [45,65,66]. In addition, the experimental studies have demonstrated a significant reduction in the level of HRNR in both the affected and unaffected skin of patients with atopic eczema, regardless of the FLG status [32,43]. Altogether, the observed association between the rs877776 variant and atopic eczema seems to be very plausible; however, whether this genetic variant could alter the expression or function of the HRNR protein has not yet been investigated. Importantly, the effects of rs877776 SNP on atopic eczema risk were independent of the FLG mutations previously described in our population, suggesting that the HRNR gene could be responsible for an additional association signal seen in EDC aside from the well-established FLG mutation.

The human filaggrin-2 (FLG2) protein is another member of the SFTP family that shares common structural features with FLG and is believed to be involved in atopic eczema pathogenesis [42,43]. The FLG2-encoding gene is located in close proximity to FLG in the EDC region and its expression is probably controlled similar to FLG at the transcriptional level [42,44,62]. FLG2 is mainly expressed in the granular keratinocytes and the lower cornified layer of the epidermis, where it is generally colocalized with FLG [43,44,46]. The exact function of FLG2 is still not fully understood; however, it is considered to be a component of CE, involved, along with FLG, in the terminal differentiation of keratinocytes and maintaining stratum corneum hydration [42,43,44,67]. Interestingly, functional studies have clearly demonstrated that decreased FLG2 expression is associated with disturbances in the process of keratinocyte differentiation and CE formation, suggesting the potential role of FLG2 downregulation in the epidermis impairment observed in atopic eczema [32,33]. Indeed, the expression level of FLG2 was found to be markedly reduced in lesional and nonlesional atopic eczema skin in comparison to healthy skin [32,46,47]. Although it needs to be confirmed, the decreased expression of FLG2 in atopic eczema patients might be explained by genetic variants in the FLG2 gene. In the systematic screening of the EDC region, Marenholz et al. identified the nonsense mutation Ser2377X in the FLG2 gene [rs12568784], which was validated by sequencing in a set of children with eczema. However, in a subsequent association analysis, this FLG2 variant was not associated with eczema in the discovery population [68]. In the present study, we provided evidence for the highly significant association of the rs12568784 [T] allele in FLG2 with atopic eczema, and the severity of the disease. Our results are in agreement with findings in a cohort of African-American children demonstrating that the rs12568784 variant in FLG2 was correlated with more persistent atopic eczema [69,70]. In contrast, no association was found between two FLG-2 SNPs (rs12568784 and rs16899374) and atopic eczema in a Brazilian population [71]. To the best of our knowledge, our study is the first report showing that a common nonsense mutation of FLG2 is associated with atopic eczema in a population of European children. However, it is worth pointing out that the effects of rs12568784 SNP on atopic eczema risk were observed only in the presence of strongly significant FLG mutations. This observation could be explained (at least partially) by the previously suggested experimental studies, regarding intermolecular cross-linking between these two proteins or by the assumption that the function of FLG2 in skin formation could be compensated by FLG [44,58,72]. However, further functional studies are required to investigate the exact biological mechanism of the effect.

Beyond indicating the association of HRNR rs877776 [C] and FLG2 rs12568784[T] with atopic eczema, our study demonstrated that these SNPs are also associated with allergic sensitization and susceptibility to the complex phenotype—asthma plus eczema. This might suggest that these risk variants, by reducing the expressions of epidermal barrier proteins, could be involved not only in atopic eczema pathogenesis but also in the mechanisms leading to atopic march and the development of other allergic phenotypes secondary to atopic eczema. This assumption derives from numerous literature data confirming the progression from atopic eczema to allergic airway diseases and suggesting that the epidermal barrier dysfunction in atopic eczema leads to increased susceptibility to allergic sensitization and, thus, contributes to the subsequent development of asthma [4,6,7,12,13,14]. In the present study, both risk alleles HRNR rs877776[C] and FLG2 rs12568784[T] provided significantly increased risks for allergic sensitizations and the effects were independent of concomitant allergic diseases. In addition, the rs877776 and rs12568784 risk variants were associated with the complex phenotype—asthma plus eczema. Due to the study design, we were unable to evaluate the association with asthma in the absence of eczema and, therefore, the same effect on the risk of asthma cannot be excluded. Nevertheless, the analysis limited to children with eczema confirmed that the observed association between HRNR and FLG2 with eczema-associated asthma is not only due to the presence of coexisting eczema. Both risk alleles, HRNR rs877776[C] and FLG2 rs12568784[T], conferred increased risks for asthma in this subgroup, indicating that these SNPs might provide genuine risks for the particular asthma phenotype occurring in the context of eczema. These results show that tested genetic variants in EDC genes could contribute, similar to FLG, to the development of more complex allergic phenotypes. Therefore, it can be assumed that genetically determined skin barrier abnormalities, such as the primary cause of atopic eczema, may be required for further progression to distinct eczema-related phenotypes with a unique pathogenic pathway. Although the exact mechanisms need to be elucidated, the experimental evidence supports the hypothesis that this distinct asthma phenotype could be induced secondary, as a downstream consequence of percutaneous priming due to the epidermal barrier disruption, increased permeability to allergens, and subsequent allergic sensitization [73,74,75,76,77]. The contribution of skin barrier impairment in allergic sensitization is now commonly accepted and was confirmed in several experimental studies [73,74,78,79,80]. The well-established association between FLG mutations and allergic sensitization suggests a role of the abnormal expression of epidermal differentiation-related proteins in the mechanism of percutaneous sensitization susceptibility [81,82,83,84,85]. Presumably, this might also be the case for other SFTP proteins. Our results could support this hypothesis as we demonstrated that HRNR and FLG2 risk variants were associated with allergic sensitization even in children without any allergic symptoms, including eczema. This is in agreement with the recent experimental study testing double-knockout mice lacking both (FLGHRNR−/−) genes, indicating that the combined deficiency of filaggrin and hornerin changes the skin barrier function leading to permeability abnormalities, epicutaneous sensitization, and allergic inflammation [86].

The same genomic organization with dense tandem arrangements and the high level of relationships among the EDC genes, their spatially and temporally coordinated expressions in the epidermis, and functional similarities of the encoded proteins increase the possibility of gene–gene interactions between tested SNPs and FLG mutations [41,42]. The distribution analysis of HRNR and FLG2 variants and the combined FLG null genotypes revealed the highest risk for atopic eczema and eczema-associated asthma in subjects who carried compounded risk factors—HRNR risk allele and FLG mutation, combined FGL2 and FLG mutations, or at least one HRNR risk allele together with at least one FLG2 risk allele. This might suggest some possible synergistic and/or multiplicative effect of all tested variants on atopic eczema and asthma in the context of eczema, as the cumulative disease risk for the combined variants was higher than that observed for these alleles separately. These findings seem to be in agreement with the observations from functional studies confirming that FLG, FLG-2, and HRNR are closely related, show coordinated expressions, and may play overlapping and perhaps synergistic roles in the proper barrier functions [33,43,72,86]. Our results highlight that, in the case of allergic diseases, such as eczema and asthma, genetic predispositions are complex and might be better explained by the cumulative effects of multiple genetic variations and the existence of gene–gene interactions.

Atopic eczema often predisposes to other allergic diseases and is a well-established risk factor for the development of asthma, particularly in cases with severe and early-onset atopic eczema [4,5,6,7]. However, not all children with atopic eczema will develop asthma and, therefore, one of the most important challenges is to define risk biomarkers to predict the course of the disease and identify patients at higher risk for progression to asthma. The linkage and association studies indicate that shared inherited susceptibility factors are mainly accountable for this increased risk of comorbid eczema and asthma [87,88]. Thus, it seemed of interest to study the application of HRNR and FLG2 variants for eczema-associated asthma prediction. In the present study, HRNR and FLG2 risk variants predicted the future development of eczema-associated asthma with a positive predictive value of approximately 50%, which improved considerably when these variants were combined with FLG mutations. In our population, 100% of children with eczema who carried one of the tested variants and at least one FLG mutation had developed eczema-associated asthma up to the age of 6. The highly positive predictive value may be due to an interaction between these EDC genes. Our findings suggest that early identification of children with atopic eczema who are genetically predisposed to epidermal skin barrier disruption might be useful in predicting the development of eczema-related asthma and could improve the efficacy of preventive measures.

Nevertheless, given the multifactorial pathogenesis of atopic eczema, it should be emphasized that increased prevalence, variation in severity of the disease, the persistent course, and subsequent progression to further atopic comorbidities cannot be adequately explained by genetic susceptibility alone [8,9]. The exact mechanisms are still the subject of intense investigation but the current understanding of atopic eczema pathogenesis and progression points toward a complex interplay between genetic backgrounds and environmental influences [53,54]. The role of environmental factors appears to be substantial as the skin is the primary defense barrier against external stimuli, and the dysfunction of the skin barrier integrity can cause a systemic breakdown of immune tolerance [10,11,14]. Various environmental exposures have been proposed to affect atopic eczema development, exacerbation, or progression in predisposed individuals. Among the best-proven risk factors are ‘Western’ diets high in sugar and trans-fatty fatty acids, broad-spectrum antibiotic exposure, small family sizes, high household education levels, and natural climatic factors, such as low ultraviolet light exposure, low relative outdoor, indoor humidity, as well as frequent using of central and electric heating [89,90,91]. Other risk factors supported by strong epidemiological data are: living in an urban area associated with specific hygiene-related exposures, increased stress, and higher exposure to traffic-related air pollution, including particulate matter, sulfur, carbon, nitrogen, and benzene components connected with urbanization and industrialization [90,91,92]. Specific external irritants, such as alkaline soaps, detergents microplastics, nanoparticles, and hard water have also been reported to play important roles in the development and aggravation of atopic eczema [93,94]. There is some evidence for the effects of maternal or postnatal tobacco exposure, long-term exclusive breastfeeding, routine childhood vaccinations, viral or bacterial infections, and farm environments; however, epidemiological data are still inconsistent [89,90,91,95]. On the other hand, some environmental exposures, such as maternal contact with farm animals during pregnancy, consumption of unpasteurized farm milk, dog ownership, and exposure to high-level endotoxins in early life have been consistently demonstrated to play protective roles in atopic eczema [89,90]. To make it more complex, recent studies indicated that environmental factors may play a role through epigenetic alterations, pointing to a crucial role of gene–environment interactions in atopic eczema pathogenesis [18,96]. However, the number of studies on gene–environment interactions in the etiology of atopic eczema is limited. In a recent systemic review, Blakeway et al. demonstrated evidence for interactions between FLG mutations and having older siblings, exposure to phthalates from household dust, urine phthalate metabolite levels, early-life exposure to cats, and water hardness, which increased the risk of atopic eczema. However, small numbers of studied individuals and lack of replication made it difficult to interpret the results [97]. Another study suggested that FLG mutations might modify the effects of smoking on the risk of asthma [98]. Based on the above, our findings should be interpreted with caution because we focused on the genetic variants as predictive factors without considering environmental factors that may interfere with the obtained results. Future research, therefore, should include genetic predisposition as well as an in-depth analysis of gene–environment interactions.

It is worth mentioning that our study design allowed us to follow the same cohort over time with repeated monitoring of risk factors and health outcomes. Another strength of our study involved using age- and sex-matched healthy controls recruited from the general population, although having a larger dataset would offer the feasibility for further extended analyses and possibly an assessment of the association between the genetic variants tested with asthma in the entire population. It is also important to consider some potential limitations of our study—it was a relatively small sample size and, as a consequence, rather low statistical power may have led us to false negative or fortuitous false positive results. Nevertheless, we were able to demonstrate statistically significant and biologically plausible effects. An interaction analysis requires a large population; hence, our results should be interpreted carefully and need to be confirmed in large cohort studies. Similarly, despite the effects of the tested risk variants on eczema-associated asthma predictions being remarkably strong, further studies are needed to confirm these findings. Another limitation of our study was the lack of a haplotype analysis, which could overcome the limitations of the single SNP analysis, increase the statistical power of the genetic analysis, and identify key SNPs or haplotypes associated with the disease. The extended haplotype analysis for all tested in our population’s SNPs, in genes located on the chromosome 1q21 region, will be performed and presented in a separate manuscript.

## 5. Conclusions

In conclusion, the present study indicates that risk variants in HRNR and FLG2 genes may contribute to atopic eczema susceptibility; for HRNR SNP, this effect seems to be independent of the well-established FLG risk alleles. In addition, our results reveal that HRNR and FLG2 variants and FLG loss-of-function mutations are candidate genes that might control the risk of atopic eczema in an interactive manner. Moreover, we demonstrated that the tested variants confer significant risks for allergic sensitization and asthma in the context of eczema, supporting the role of EDC genes and epidermal barrier dysfunction in the development of distinct eczema phenotypes predisposed to progression towards other atopic diseases. In children with eczema, these genetic variants may also be considered, along with FLG mutations, as predictive biomarkers for eczema-associated asthma. Further confirmation of our findings and experimental studies will be required to better understand the functional implications of these genetic variants in the pathogenesis of atopic eczema and subsequent atopic march, which may contribute to the development of effective prevention strategies.

## Figures and Tables

**Table 1:** Characteristics of the study group.

Variable	Atopic Eczema	Control
Age at the time of recriutment, month (mean ± SD)	13.6 ± 6.7	15.9 ± 5.6
Gender (male/female)	63/40	44/41
Allergic sensitization (%)	55 (53.4%)	11 (12.9%)
Asthma (%)	28 (27.2%)	0
SCORAD Mild Moderate Severe	79 (76.7%) 24 (23.3%) 0	0 0 0
Atopic hereditary (%)	57 (55.3%)	0
Serum Total IgE, IU/mL, geometric mean, 95% CI	24.6 (27.8 ÷ 53.4)	17.7 (14.3 ÷ 22.2)
HRNR rs877776 GG GC CC	60 (58.3%) 37 (35.9%) 6 (5.8%)	66 (77.6%) 15 (17.7%) 4 (4.7%)
FLG2 rs12568784 GG GT TT	74 (71.8%) 20 (19.5%) 9 (8.7%)	72 (84.7%) 8 (9.4%) 5 (5.9%)
Combined FLG genotype (R501X, 2282del14, R2447X, S3247X) Normal Null	89 (86.5%) 14 (13.5%)	83 (98%) 2 (2%)

**Table 2:** Associations between the HRNR rs877776 genotype and allergic diseases.

Phenotype	Total *n*(%)	HRNR rs877776 Genotype Status			
		GG (%)	GC + CC (%)	*p*-Value	OR (95% CI)
A: Whole study population					
Eczema	103/188 (54.8%)	60/126 (47.6%)	43/62 (69.4%)	*p* = 0.005	2.48 (1.25 ÷ 4.99)
Eczema Mild Moderate	60/103 (58.3%) 43/103 (41.7%)	34/60 (56.7%) 26/60 (43.3%)	26/43 (60.5%) 17/43 (39.5%)	----- *p* = 0.840	1.0 Reference 1.17 (0.48 ÷ 2.80)
Asthma plus eczema	28/188 (14.9%)	11/126 (8.7%)	17/62 (27.4%)	*p* = 0.002	3.94 (1.71 ÷ 9.10)
Allergic sensitization	66/188 (35.1%)	37/126 (29.4%)	29/62 (46.8%)	*p* = 0.023	2.14 (1.07 ÷ 4.16)
Polysensitization (Sepc. IgE > 1)	19/188 (10.1%)	11/126 (8.7%)	6/62 (12.9%)	*p* = 0.039	2.09 (1.11 ÷ 7.69)
B: Eczema group					
Asthma	28/103 (27.2%)	11/60 (18.3%)	17/43 (39.5%)	*p =* 0.024	2.91 (1.19 ÷ 7.13)
Allergic sensitization	55/103 (53.4%)	27/60 (45.0%)	28/43 (65.1%)	*p* = 0.044	2.28 (1.02 ÷ 5.11)

Allergic sensitization: positive Spec. IgE to at least 1 of tested allergen; Polysensitization: positive Spec. IgE to more than 1 tested allergen. The control group comprises all individuals, who do not belong to any of the disease group.

**Table 3:** Associations between the FLG2 rs12568784 genotype and allergic diseases.

Phenotype	Total *n*(%)	FLG2 rs12568784 Genotype Status			
		GG (%)	GT + TT (%)	*p*-Value	OR (95% CI)
A: Whole study population					
Eczema	103/188 (54.8%)	74/146 (50.9%)	29/42 (69.0%)	*p* = 0.035	2.17 (0.99 ÷ 4.81)
Eczema Mild Moderate	60/103 (58.3%) 43/103 (41.7%)	50/60 (83.3%) 24/60 (40.0%)	10/43 (23.3%) 19/43 (47.5%)	----- *p* = 0.002	1.0 Reference 3.95 (1.46 ÷ 10.88)
Asthma plus eczema	28/188 (14.9%)	13/146 (8.9%)	15/42 (35.7%)	*p* = 0.000	5.68 (2.43 ÷ 13.3)
Allergic sensitization	66/188 (35.1%)	41/146 (28.1%)	25/42 (59.5%)	*p* = 0.000	3.76 (1.74 ÷ 8.19)
Polysensitization (Sepc. IgE > 1)	19/188 (10.1%)	9/146 (6.2%)	10/42 (23.8%)	*p* = 0.001	2.88 (1.33 ÷ 6.26)
B: Eczema group					
Asthma	28/103 (27.2%)	13/74 (17.6%)	15/29 (51.7%)	*p* = 0.001	5.03 (1.95 ÷ 12.9)
Allergic sensitization	55/103 (53.4%)	35/74	20/29	*p* = 0.047	2.47 (0.99 ÷ 6.15)

Allergic sensitization: positive Spec. IgE to at least 1 of tested allergen; Polysensitization: positive Spec. IgE to more than 1 tested allergen. The control group comprises all individuals, who do not belong to any of the disease group.

**Table 4:** Atopic eczema risk after stratification for FLG mutations.

Genotype	FLG Normal *p*-Value OR (95% CI)	FLG Null *p*-Value OR (95% CI)
HRNR rs877776		
Dominant model GG vs GC+CC	*p* = 0.007 2.47 (1.21 ÷ 5.06)	*p* = 0.180 2.60 (0.69 ÷ 9.27)
Allele model G vs C	*p* = 0.011 2.04 (1.13 ÷ 3.70)	*p* = 0.005 3.48 (1.40 ÷ 8.62)
FLG2 rs12568784		
Dominant model GG vs GT+TT	*p* = 0.552 1.36 (0.58 ÷ 3.22)	*p* = 0.016 1.66 (0.96 ÷ 1.66)
Allele model G vs T	*p* = 0.908 1.04 (0.50 ÷ 2.15)	*p* = 0.015 1.44 (0.99 ÷ 1.40)

**Table 5:** Interaction between HRNR rs877776 and FLG2 rs12568784 and the FLG mutations in atopic eczema.

Genotype Combinations	Atopic Eczema *n(%)*	Control *n(%)*	*p*-Value	RR (95% CI)	
HRNR rs877776 GG FLG mutation (−)	52 (50.5%)	64 (75.3%)	-----	1.00 (Reference) -	RERI = −0.02; AP = −0.008; S = 0.02 ratio of RRs = 0.85 *p* = 0.9978
HRNR rs877776 GC+CC FLG mutation (−)	37 (35.9%)	19 (22.3%)	*p* = 0.010	1.47 (1.08 ÷ 1.92)	
HRNR rs877776 GG FLG mutation (+)	8 (7.8%)	2 (2.4%)	*p* = 0.047	1.78 (0.94 ÷ 2.22)	
HRNR rs877776 GC+CC FLG mutation (+)	6 (5.8%)	0	*p* = 0.010	2.23 (1.12 ÷ 2.23)	
FLG2 rs12568784 GG FLG mutation (−)	71 (68.9%)	70 (82.3%)	-----	1.00 (Reference) -	RERI = 0.64; AP = 0.32; S = 2.88 ratio of RRs = 1.45 *p* = 0.9980
FLG2 rs12568784 GT+TT FLG mutation (−)	18 (17.5%)	13 (15.3%)	*p* = 0.552	1.15 (0.75 ÷ 1.58)	
FLG2 rs12568784 GG FLG mutation (+)	3 (2.9%)	2 (2.4%)	*p* = 1.000	1.19 (0.33 ÷ 1.88)	
FLG2 rs12568784 GT+TT FLG mutation (+)	11 (10.7%)	0	*p* = 0.001	1.98 (1.31 ÷ 1.98)	
HRNR rs877776 GG FLG2 rs12568784 GG	49 (47.6%)	60 (70.6%)	-----	1.00 (Reference) -	RERI = −0.34; AP = −0.21; S = 0.63 ratio of RRs = 0.69 *p* = 0.4461
HRNR rs877776 GC+CC FLG2 rs12568784 GG	25 (24.3%)	12 (14.1%)	*p* = 0.022	1.50 (1.04 ÷ 1.98)	
HRNR rs877776 GG FLG2 rs12568784 GT+TT	11 (10.7%)	6 (7.1%)	*p* = 1.191	1.44 (0.82 ÷ 2.01)	
HRNR rs877776 GC+CC FLG2 rs12568784 GT+TT	18 (17.4%)	7 (8.2%)	*p* = 0.025	1.60 (1.05 ÷ 2.08)	

**Table 6:** Interaction between HRNR rs877776 and FLG2 rs12568784 and the FLG mutations in eczema-associated asthma.

Genotype Combinations	Eczema-Associated Asthma *n(%)*	Control *n(%)*	*p*-Value	RR (95% CI)	
HRNR rs877776 GG FLG mutation (−)	10 (35.7%)	64 (75.3%)	-----	1.00 (Reference) -	RERI = 2.19; AP = 0.29; S = 1.52 ratio of RRs = 0.76 *p* = 0.9978
HRNR rs877776 GC+CC FLG mutation (−)	12 (42.8%)	19 (22.3%)	*p* = 0.004	3.01 (1.36 ÷ 6.60)	
HRNR rs877776 GG FLG mutation (+)	2 (7.2%)	2 (2.4%)	*p* = 0.374	2.46 (0.12 ÷ 7.68)	
HRNR rs877776 GC+CC FLG mutation (+)	4 (14.3%)	0	*p* = 0.001	7.40 (2.48 ÷ 7.40)	
FLG2 rs12568784 GG FLG mutation (−)	10 (35.7%)	70 (82.3%)	-----	1.00 (Reference) -	RERI = 0.36; AP = 0.045; S = 1.05 ratio of RRs = 0.43 *p* = 0.9982
FLG2 rs12568784 GT+TT FLG mutation (−)	12 (42.8%)	13 (15.3%)	*p* = 0.000	3.84 (1.74 ÷ 8.20)	
FLG2 rs12568784 GG FLG mutation (+)	3 (10.7%)	2 (2.4%)	*p* = 0.024	4.80 (1.16 ÷ 8.84)	
FLG2 rs12568784 GT+TT FLG mutation (+)	3 (10.7%)	0	*p* = 0.003	8.00 (2.13 ÷ 8.00)	
HRNR rs877776 GG FLG2 rs12568784 GG	6 (21.4%)	60 (70.6%)	-----	1.00 (Reference) -	RERI = −4.52; AP = −0.69; S = 0.77 ratio of RRs = 0.32 *p* = 0.3350
HRNR rs877776 GC+CC FLG2 rs12568784 GG	7 (25.0%)	12 (14.1%)	*p* = 0.007	4.05 (1.34 ÷ 11.1)	
HRNR rs877776 GG FLG2 rs12568784 GT+TT	5 (17.9%)	6 (7.1%)	*p* = 0.007	5.00 (1.47 ÷ 14.3)	
HRNR rs877776 GC+CC FLG2 rs12568784 GT+TT	10 (35.7%)	7 (8.2%)	*p* = 0.000	6.47 (2.52 ÷ 16.2)	

**Table 7:** The predictive values of risk factors (HRNR rs877776, FLG2 rs12568784, FLG mutations) for the eczema-associated asthma phenotype.

Predictor	Sensitivity, % (95% CI)	Specificity, % (95% CI)	Positive Predictive Value (PPV), % (95% CI)	Negative Predictive Value (NPV), % (95% CI)
HRNR rs877776	57.14 (37.18 ÷ 75.54)	77.65 (67.31 ÷ 85.97)	45.71 (32.59 ÷ 58.37)	84.62 (77.94 ÷ 89.54)
FLG2 rs12568784	53.57 (33.87 ÷ 72.49)	84.71 (75.27 ÷ 91.60)	53.57 (38.59 ÷ 67.93)	84.71 (78.65 ÷ 89.28)
FLG mutations	30.00 (11.89 ÷ 54.28)	97.65 (91.76 ÷ 99.71)	75.00 (33.09 ÷ 93.34)	85.57 (81.62 ÷ 88.78)
HRNR rs877776 FLG mutations	28.57 (8.38 ÷ 58.10)	100.0 (94.40 ÷ 100.0)	100.0 (68.9 ÷ 100.0)	86.49 (82.13 ÷ 89.91)
FLG2 rs12568784 FLG mutations	23.08 (5.04 ÷ 53.81)	100.0 (94.87 ÷ 100.0)	100.0 (69.2 ÷ 100.0)	87.50 (83.86 ÷ 90.41)

## Data Availability

The data presented in this study are available upon request from the corresponding author.
